# Towards more equitable education: *meeting health and wellbeing needs of newly arrived migrant and refugee children—perspectives from educators in Denmark and Sweden*

**DOI:** 10.1080/17482631.2020.1773207

**Published:** 2020-12-09

**Authors:** Claire Mock-Muñoz de Luna, Alexandra Granberg, Allan Krasnik, Kathrine Vitus

**Affiliations:** a Danish Research Centre for Migration, Ethnicity and Health; Section for Health Services Research, Department of Public Health, University of Copenhagen, Copenhagen K, Denmark; b Department of Social and Policy Sciences, University of Bath, Bath, UK; c School of Global Health, University of Copenhagen, Copenhagen, Denmark; d Danish Research Centre for Migration, Ethnicity and Health; Section for Health Services Research, Department of Public Health, University of Copenhagen, Copenhagen, Denmark; e Institute for Sociology and Social Work, University of Aalborg, Aalborg, Denmark

**Keywords:** Migrant and refugee children, health inequalities, equity in education, migrant education policy, educators, Scandinavia

## Abstract

*Purpose* - In 2015, Scandinavia experienced the arrival of many refugee children. Research has documented a higher prevalence of mental health problems among refugee compared to non-migrant children. Education and schools play an important role in the health and wellbeing of children, especially those who are vulnerable, and equity in education may help combat social and health inequalities. This study investigated educators’ views on the health and wellbeing needs of migrant children in Copenhagen, Denmark, and Malmö, Sweden, and how schools may address these issues. *Methods* - We carried out 14 semi-structured interviews with education professionals in both cities and conducted a thematic analysis inspired by the Street Level Bureaucracies theory. *Results* - Most interviewees recognized NAMR pupils had specific migration-related needs but some expressed being unable to cope with more complex issues due to a lack of vital health and wellbeing services within schools. Recent policies in Denmark further devolved migrant education to municipalities; while in Sweden new policies centralized and standardized procedures. *Conclusion* - To summarize, educational leaders and staff we interviewed in both countries felt that the lack of resources, professional training, standardized procedures and accountability measurement, together with inflexible systems, inhibited them from providing equitable education, thus possibly reinforcing migration-related health inequalities.

## Introduction

This study investigates the experiences and perceptions of educators regarding the health and wellbeing needs of newly arrived refugee children in Copenhagen, Denmark, and Malmö, Sweden, and the role of schools in addressing these needs. Education in Denmark and Sweden serves as an integral part of the political system constituting the Nordic Welfare system, promoting traditional social democratic values, such as inclusion, participation, citizenship, and equality (Telhaug et al., 2006). Universal strategies promoting equality in education are considered a vital instrument in levelling social inequalities (Galloway et al., 2015).

Recent migratory flows to Denmark and Sweden included a significant number of refugee children. In 2015, arrivals seeking asylum in Sweden peaked at over 156,000 and 20,000 in Denmark, mainly originating from Syria, followed by Afghanistan, Iraq, Somalia, and Eritrea—all regions mired in violent conflict (Bunar, 2017). Of these, between 30% and 44% were children including many unaccompanied asylum seeking children (UASC) (Bunar, 2017; Nilsson & Bunar, 2016; Nordic Council of Ministers, 2016).

These migratory flows have impacted Scandinavian education systems to varying degrees: in 2015 the number of migrant pupils entering compulsory schooling in Sweden was 49,500, or 5% of all students. In Denmark, in 2016, 8% of the almost 670,000 compulsory school pupils were descendants, and over 3% or approximately 80,000 pupils were immigrants (Linnemann Johansson, 2017).

Research on refugee and asylum seeking children, in particular, i.e., those who have been granted temporary protection and those who are awaiting a decision regarding their permission to stay, has documented an increased susceptibility to physical and mental health problems because of their migration experience (Barghadouch et al., 2018; Fazel et al., 2012; Hjern & Kadir, 2018; Montgomery, 2010; Montgomery & Foldspang, 2008). They may have been exposed to migration-related risk factors in the country of resettlement that negatively impact health and wellbeing, and impede their learning. Risk factors include poverty, marginalization, discrimination, and since 2015, in Denmark especially, increasingly restrictive immigration policies, e.g., restrictions to family reunification (Bech et al., 2017; Fazel et al., 2012).

Research findings like those presented above may explain the ubiquity of “trauma discourse” associated with refugees and their experiences. Focusing on pre-migratory experiences tends to obscure extenuating circumstances experienced by many in the destination country. Restrictive immigration policies, prolonged stays at reception centres, added requirements for family reunification, uncertainty, and the threat of deportation are known stressors negatively impacting the health and wellbeing of many asylum seekers and refugees (Jervelund et al., 2008). Poverty, discrimination, and isolation experienced by many migrants in general are also common features of life in the destination country. Pushing back against this “trauma discourse”, and in line with our position, some studies invoke a “strength-based” approach to migrant and refugee children, recognizing their courage and resilience in rebuilding their lives and reclaiming their place in society (Block et al., 2014; Pinson & Arnot, 2007; Rutter, 2006). These studies emphasize that education systems, to abide by notions of equality and equity in education, need to engage with these children in a child-centred way, identifying and harnessing their strengths while being conscious of- and addressing their vulnerabilities. Thus, in addition to its fundamental role in preparing individuals for productive and fulfiling lives through learning and socialization, research shows that the presence of migrant and refugee children requires education systems to reflect the complexity and diversity of needs of this group (Arnot et al., 2009).

Finally, schools are known as potential public health settings for children, especially vulnerable ones, shown to improve health in the short term and promote health equity in the long term (Hahn & Truman, 2015). However, schools may also act to further marginalize already vulnerable children and to reinforce or deepen social stratification. For NAMR children especially, education and schools may potentially expose them to the support of school health services and caring adults well positioned to identify symptoms of mental health problems and make referrals to appropriate services at an early stage of resettlement (Barghadouch et al., 2016; Block et al., 2014; Borsch et al., 2019; Fazel et al., 2014; Pastoor, 2015; Rutter, 2006). Participation in education represents meaningful activity in a safe environment, offering opportunities to rebuild social networks and participate in society, across cultures, fostering sense of coherence, agency and hope—processes closely linked to health and wellbeing (Borsch et al., 2019; Jarlby et al., 2018; Krause, 2011; Mayer & Boness, 2011).

In Scandinavia, universal access to education for all children who are residents is one of the ways in which equality in education is operationalized. In practice, access varies from country to country and is tied to immigration status (Tørslev & Børsch, 2017). *Equal access* is distinct from *equity in education*, i.e., how education and schools facilitate conditions for children to thrive, regardless of background (Castelli et al., 2012). In Scandinavia, educational outcomes are not equal among different pupil groups, suggesting that the design and structure of education provision may not be equitable for all pupils by failing to adequately meet diverse needs and ensuring equitable access to opportunities offered by education.

### Analytical framework

Nonetheless, policy “on paper” often differs from the interpretation and implementation of these policies “on the ground”, especially in Scandinavia’s highly decentralized education systems. Educators, including teachers and headmasters, are at the frontline of education policy implementation, and therefore positioned to give valuable insight into how they interpret and enact these policies, and the factors influencing their practices (Lipsky, 1972). A theoretical framework that gives analytical, methodological and normative insight into the lived experiences of public servants on the frontline of policy implementation is the theory of “Street Level Bureaucracies” (SLB) (Lipsky, 2010). Michael Lipsky coined the term in 1971 to refer to the contexts where citizens “experience directly the government they have implicitly constructed (Lipsky, 1972).” (Brodkin, 2015). This study takes inspiration from Lipsky’s approach and focuses on teachers, who, as “street-level bureaucrats” interpret and implement policies in their specific municipal and school contexts, characterized by, among others, rules, availability of resources, societal and political pressures, and demands from municipal and central government (Lipsky, 1972).

In this study, we focus on how the structures and organizational conditions, shaped by policies, in turn, shape the coping practices of teachers and headmasters. While the analysis addresses the impact of policies on street level organizations’ (SLO) practices what Brodkin categorizes as an SLO “policy study” (Brodkin, 2011), the theoretical presumption from the SLO perspective is that the real-life practices of public servants have real-life outcomes for their clients (Brodkin, 2011). Thus, teachers’ and headmasters’ coping strategies and behaviours condition to what extent and how NAMR children’s health and wellbeing are addressed in school.

In anticipation of the increased number of refugee children entering their education systems, both Denmark and Sweden introduced new education policies concerning schools’ specific obligations to NAMR children in compulsory school. We hypothesized that these new education policies for NAMR students would take into account the potentially elevated incidence of psychosocial distress and poor wellbeing among these pupils, as well as educators’ role in addressing these issues. Educators in Copenhagen, Denmark, and Malmö, Sweden, may be able to reveal what these policies look like “on the ground” and shed light on factors that hinder or foster the ability of professionals to meet the needs of their pupils.

To this end, we ask the following research questions: 1) How did schools in Copenhagen and Malmö structure their provision of education for NAMR students following the introduction of policy changes?; 2) what do educators perceive and experience are the health and wellbeing needs of newly arrived migrant and refugee pupils?; 3) are they able to address these health and wellbeing needs within the national policy framework, and how?

### Terminology

In this study, we use the term **“newly arrived migrant and refugee” (NAMR)** pupils/students or children to refer to this heterogeneous group, consisting of all sorts of educational, cultural, religious, and socioeconomic backgrounds, and diverse migration trajectories. Our choice of terminology is based on recent definitions by the International Organization for Migration (IOM) and the World Health Organization (WHO). IOM uses “migrant” as an umbrella term covering individuals who move away from their usual place of residence, and including all categories of migrants, regardless of legal status, or whether they migrate internally or across borders (International Organization for Migration, 2018). The WHO prefers the terms “migrants and refugees” highlighting that refugees feature as a subgroup of migrants in terms of potential health risk factors (World Health Organization, 2019).

We adopt the World Health Organization (WHO) definition of **health**, referring to the absence of illness and a range of other elements constituting a healthy life, e.g., positive mental health, wellbeing and quality of life (World Health Organization, 1948). **Wellbeing**—a debated concept given the methods for measuring outcomes, i.e., the use of self-reporting—is often considered in relation to mental health, and is relevant, especially in the context of schools, where much of a child’s social, cognitive and emotional development takes place (Ben-Arieh et al., 2014) (Konu & RimpelÄ, 2002). Wellbeing facilitates children’s full participation and enjoyment of the benefits and opportunities offered by education and school attendance (O’Connor et al., 2019). “Wellbeing” in this study refers to the social, emotional, cognitive and behavioural welfare that ideally characterizes a child’s daily life, allowing them to participate fully and reach their potential (Ben-Arieh et al., 2014; Hunner-Kreisel & März, 2019).

## Methods and materials

During the Spring of 2018, using purposeful sampling in Copenhagen, Denmark and Malmö, Sweden, we identified potential participants among teachers who work with newly arrived pupils and headmasters at schools with a high proportion of migrant and refugee pupils. (Mason, 2002). Purposeful sampling is usually employed to ensure the “identification and selection of information-rich cases for the most effective use of limited resources”, and may hamper generalizing findings as it often neglects the heterogeneity of population groups sampled (Palinkas et al., 2015).

To identify these potential participants, first we consulted municipal websites listing all compulsory state primary and lower secondary level schools in Malmö, Sweden and Copenhagen, Denmark, attended by children aged 7–15. Upper secondary level schools were not within the scope of this study. Most unaccompanied asylum seeking children enter the Danish and Swedish education systems at this level, i.e., aged 16–19, and we recognize that they form an important group numerically, facing unique challenges and circumstances, requiring different education and social care support arrangement. The complexity of these issues would require a specially focused study.

Our second recruitment strategy was to contact municipal departments responsible for education in each city to identify schools that received large numbers of migrant pupils in the past and had experience in working with this pupil group. We then introduced the project via emails and follow-up phone calls to heads of schools, inviting them and/or teachers of Swedish as a Second Language (SSL) and Danish as a Second Language (DSL) at their school to participate in the research. We continued to recruit until we achieved data saturation, i.e., when interviews stopped yielding new information.

Our sample is represented in the table (Table I):

**Table I.** Sample of study participants

City, Country	Teachers	Headmasters	Municipal staff at reception unit/pilot programme	Gender	Schools represented	Total participants
Copenhagen, Denmark	4	2	1	4 female, 3 male	3	7
Malmö, Sweden	3	2	2	7 female	4	7

The municipal workers in Malmö included a manager and a development secretary (utvecklingssekreterare) at the municipal reception department’s (Mottagningsenheten) “language centre” (Språkcentralen), responsible for the assessment and introduction of NAMR children into the Malmö school system. In Copenhagen, we interviewed the Municipal programme manager responsible for the development and implementation of the New in Copenhagen initiative. The municipal workers provided relevant contextual information about policies and procedures in both settings. Of the seven teachers, six were female and one male, one of the headmasters was male and three were female, and one of the municipal workers was male while the other two were female.

We supplemented semi-structured interview data with observation to provide context, attending three professional events in Malmö and Copenhagen, including a two-day conference in April 2018 for teachers specializing in language teaching for newly arrived migrant pupils in Scandinavia. The conference, organized by the Municipality of Malmö and FLIN (*Flerspråklighed i Norden*/Multi-lingualism in the Nordic Countries), and hosted by Malmö University, was attended by the lead author. Additional observation took place during two network meetings for teachers of NAMR pupils in Copenhagen, providing context both for the interview question guide, and background to the findings.

Interviews were conducted in both countries from April to October 2018. The lead researcher had limited language skills in Swedish, so, where possible, interviews in Malmö were conducted in English. Interviews in Denmark were conducted in Danish, by the lead author and last co-author (KV) with follow-up questions and clarification in English. The Danish interviews were transcribed by a student assistant at the University of Copenhagen, and the Swedish interviews were transcribed by the second listed co-author (AG), who also conducted analysis of the Swedish interviews, as described in more detail below.

### Analytical strategies

Our interview analysis combines deductive and inductive analysis. Recurrent emergent themes were coded based on synergies between the questions guiding this research, inspired by the SLB theoretical framework, and the empirical material (Brodkin, 2015; Lipsky, 2010). Concepts from the SLB approach such as “coping” and “discretion” are gleaned from the interviews through descriptions by the interviewees of the constraints (structural, organizational, resources), demands (organizational, governmental), and resource availability characterizing daily work (Baviskar & Winter, 2017; Brodkin, 2011). These concepts highlight educators’ role in meeting pupils’ complex needs, and dilemmas they face as agents of the state who are well positioned to identify and address health and wellbeing concerns of pupils. Subsequent readings of the interviews were inductive in that we looked for additional themes that emerged from the data but were outside of the SLB framework that led out deductive analysis, i.e., marginalization or invisibility of introductory classes within mainstream schools in participating Copenhagen schools. We then embedded the emerging themes in existing theoretical frameworks.

Informed by the SLB framework’s focus on the structural and individual factors shaping educators’ practices, we organized the data in three categories: *Organizational* and *structural* features of education for NAMR students; *Problematization*, or how educators view NAMR children, their health and wellbeing in the context of education and school; and *Practices* facilitating/hindering the promotion of health and wellbeing in NAMR children. The category *organizational and structural features* reflects the individual and structural resource availability that, according to the SLB framework, facilitates or constrains the choice of discretionary practices educators implement in their encounters with pupils. *Problematization* captures how educators perceive the demands placed on them as professionals and agents of the state, in light of the complex needs of NAMR pupils and different expectations of pupil achievement. *Practices* reflect the previous categories which act as determinants. The theme of *Marginalization and invisibility* of introductory classes in Copenhagen schools emerged from the Danish data and is discussed separately from the SLB framework as it was perceived central to the experiences of educators interviewed in Copenhagen.

The data sets in both settings were initially coded and analysed as separate cases given the divergent policy approaches, in order to fully appreciate the contextual qualities, and characteristics of each data set (Saldaña, 2015). This enabled us to study how these differences shape educators’ practices in each setting. Initially, the lead author coded and analysed the Copenhagen interviews according to the categories of information dictated by the research questions, and AG coded and analysed the Malmö interviews in the same way. The main categories and sub-categories to emerge from each setting were then compared in order to identify possible areas of overlap and difference (Saldaña, 2015). Themes to emerge from the interviews were also noted and compared.

## Findings

In the sections below, we first present a brief outline of the education policy context for NAMR students in both settings, followed by our findings. In Copenhagen, our findings reflected the recent introduction of legislation allowing municipalities to design alternative provision for NAMR pupils, while in Malmö study participants referred to national level policies and guidelines applying equally to all municipalities in Sweden. To answer our research questions, we organized findings according to conceptual categories derived from the SLB framework presenting different factors shaping professional interactions, i.e., organizational structure of education for NAMR students (Question 1); problematizations informing educators’ perspectives of their “clients” and clients’ problems (Question 2); and the resulting practices characterizing educators’ interactions with pupils (Question 3).

### The case of Copenhagen, Denmark

#### Policy context

Following the 2015 increase in migratory flows, the Danish Ministry of Education proposed several laws to expand the existing framework for the reception of immigrant students in the education system, first by increasing the maximum number of students allowed in introductory classes from 12 to 15, and the number of grade levels covered from three to five, and second, by allowing municipalities to set up alternative or supplementary provision to the existing framework for reception classes, including special support for students with learning disabilities or mental health problems. The new framework does not fall under the Danish Education Act, which means there is limited scope for ensuring equal standards and aim for all students, and legislative accountability (Education, 2016a). In 2016, the municipality of Copenhagen introduced a pilot scheme for the education of NAMR children called the New in Copenhagen Model (Ny i KBH), to be implemented over a three-year period (20). The model, according to municipal documents, aims to address the gap in educational outcomes between migrant and Danish born pupils, by improving and speeding up Danish language learning and integration into Danish society. The means for achieving these aims include 1) direct integration into mainstream education for pupils who are assessed as academically able; 2) shortening the time NAMR pupils spend in introductory classes; 3) transitioning them sooner into mainstream education, and 4) ensuring that more young pupils start directly in mainstream classes (20). Key measures include assessment of all NAMR pupils’ academic background and skills, and a reduction in the number of introductory classes in Copenhagen schools. The funding framework for introduction classes remains the same, while resources have been set aside for continued language support for each pupil who is directly integrated into mainstream education. As of 2002, mother-tongue education in Denmark is only available to pupils from the EU, EEA, Faroe Islands and Greenland (13).

#### Organizational aspects of education for newly arrived migrant students

Of the four Copenhagen schools that participated in our study, three had introductory classes, while one didn’t. This last school had received a small number of NAMR children who had been deemed capable of being directly integrated into mainstream education.

Interviewees in Copenhagen described how the New in Copenhagen (NiC) Model introduced changes to the organization and delivery of education for NAMR students. The NiC Model was piloted only in Copenhagen, under the 2017 *Act on Special Municipal Programmes for Certain Immigrant Children and Youth* which allowed municipalities to set up alternative educational provision for NAMR students (Danish Ministry Education, 2016c). Participating teachers and one school leader indicated that the comprehensive assessment introduced through NiC signalled a shift from a deficits-based approach to one focusing on strengths, skills and knowledge children bring with them. Nevertheless, some educators found that the screening reports detailing NAMR pupils’ educational background and skills were lengthy, of varying quality and not always useful.

The NiC model’s inclusion of funding for additional classroom language support for pupils transitioning from introductory- to mainstream classes was perceived with caution by teachers because this funding was not earmarked for that purpose and could be allocated according to school leader’s discretion. This was confirmed by one of the headmasters we interviewed.

Furthermore, teachers said that limited resources and changes to the structure and class sizes made it increasingly challenging to conduct differentiated teaching, adding to the pressure to speed up NAMR pupils’ transition into mainstream classes. For pupils who are older upon arrival, these difficulties were further compounded. As one interviewee expressed put it, in reference to the competing pressures and lack of clarity on the allocation of continued language support:

I have some cases, where I have this child, and I look into my crystal ball and I can see that they’ll never be assessed as ready for secondary education. They’ll never be ready to join a normal ninth grade. If I have to transition them into mainstream education after two years, what they’ll get is failure and defeat. I can’t … It’s such a shame. It’s professional neglect. I don’t think such cases are unique. (Teacher 6)

Regarding the availability of school resources for addressing complex needs of NAMR pupils, all interviewees described the significance of access to professional support, e.g., school psychologists, and regular resource meetings to discuss pupils’ needs and support strategies. However, a common theme that was brought up by participating teachers was the lack of targeted support, i.e., professionals specifically attached to the introductory classes, who could address the special issues facing NAMR pupils and families without entering the slow system of universal child mental health services or special education services.

Regarding external resources, in one participating school with no introductory classes, ongoing support for language teaching was provided by partnered teacher training institutions delivering extra training to all teachers at the school. Municipal support was also mentioned by most interviewees, including municipal consultants, telephone hot-line, and professional training on the roll-out of the NiC Model. The organizational structure described by interviewees was greatly influenced by the different dimensions of the NiC Model, i.e., screening, resources, class sizes, and in line with SLB, influenced the ways in which interviewees perceived their ability to meet the needs of NAMR pupils.

#### Problematizations: teachers vs. headmasters’ views on the situation and needs of NAMR pupils

Teachers and headmasters in Copenhagen framed NAMR pupils and their health and wellbeing in contrasting ways: both headmasters viewed the needs of NAMR pupils as no different from those faced by Danish-born children, and framed the main issue as poor language skills and socio-economic deprivation. In contrast, all participating teachers expressed that these children and their families had migration-related needs that went beyond language learning needs, e.g., safety, stability, and to feel included. One leader working at a school that directly integrates NAMR pupils emphasized that the school was geared towards language acquisition, pupil wellbeing and the identification of social problems in general. She added that despite the school’s location in a disadvantaged area with many migrants, there was no expertise or focus on the particular challenges faced by some refugee pupils:

Some of what takes up a lot [of our attention] in general are language-weak children, so you might say that we have knowledge and expertise […] then there are the issues about what they [NAMR] carry. We don’t have any specific ways of tackling that. But then again, our school hosts a lot of children who carry heavy burdens. (School Leader 1)

The school leader said she didn’t know how many pupils at her school had a refugee background, and expressed reluctance to ask families about their background. Yet, the same interviewee acknowledged that schools and educators needed the expertise to identify children with migration-related difficulties such as “war-related trauma.” This reference to “war-related trauma” reflects an over-simplification of the migration-specific issues some NAMR pupils may face, thus overlooking other migration-related factors that may impact on a NAMR pupil’s wellbeing. As stipulated by our analytical framework, SLB may, in effect, rewrite policy through their interpretation and implementation, choosing which aspects to emphasize or sideline. The school leader thus reflected the fact that it would be more desirable for teachers to have the competencies to recognize and support different needs NAMR might have, and for schools to include features that increase the chances for students with trauma to benefit educational opportunities equally and inclusively.

The educators we interviewed in Copenhagen all concurred that health and wellbeing are a pre-requisite for being able to fully enjoy the opportunities offered by education and school settings, and vice-versa. However, the participating schools varied in the extent to which they recognized the specific needs of NAMR pupils, and in their level of expertise on how to identify and address these needs.

#### Practices: educators’ strategies for addressing pupils’ health and wellbeing needs

Practices promoting the health and wellbeing of NAMR pupils were described by all the educators we interviewed, despite the lack of explicit expectations or demands to do so in policy. However, practices were shaped by the way in which educators problematized NAMR pupils’ health and wellbeing needs, the structural features determining resources available to support these pupils, and by how integrated or marginalized the introduction classes were within the greater school context. The practices described exemplify the concept of “coping” described in the SLB framework.

Most practices related to addressing the health and wellbeing needs of NAMR pupils were initiated and implemented by the teachers themselves, while other practices, thanks to the insistence of introduction class teachers, became integral to schools’ inclusion strategies. These practices aimed to counteract the marginalization of introductory classes, their pupils and teachers, within the school setting. One interviewee described that NAMR pupils and introductory classes as “invisible” because they were taught separately and there were no occasions for interacting with the wider school. As a result, this interviewee introduced school-wide events celebrating shared occasions, e.g., Christmas and end-of-school-year. Another teacher described setting up a “sponsor” or “buddy” system pairing NAMR pupils with a native peer of the same age to familiarize them with the new school. These initiatives were mostly ad hoc and dependent on individual initiatives.

Some teachers and headmasters in Copenhagen expressed that uncertainty about their role in identifying and addressing non-academic challenges faced by NAMR pupils shaped their practices. For instance, one teacher described a student who told her about experiences in Syria before he and his family fled, at inopportune times, i.e., the middle of P.E. (physical education) class. Unable to deal with it in the ongoing lesson, the teacher had taken no further action, leaving her feeling unsure about whether she should have done more.

Another teacher described how asking a parent about their journey to Denmark in the first introductory meeting, prompted a tearful account of the family’s traumatic journey across the Mediterranean. The teacher said that while on this occasion the information she elicited was useful and important for understanding a pupil’s background, she nonetheless felt uncertain about the professionalism of such a practice, and apprehensive about repeating it in subsequent meetings. As a matter of fact, all interviewees seemed to hold the opinion that a school should be able to cope with the mental health and wellbeing challenges of all pupils. However, on an individual level, they also expressed being ill-prepared to deal with the psychosocial challenges that some NAMR pupils experienced, and that their teacher training had not covered:

These examples of professional inactivity or avoidance, brought on by a lack of appropriate in-school services, and gaps in training, support notions of Street Level Bureaucrats’ coping in the face of stressful working conditions (Brodkin, 2015).

### The case of Malmö, Sweden

#### Policy context

The Malmö schools participating in our study faced a different migration and policy context than that in Copenhagen, due to a proportional number of migrants it received in 2015, and because the education policies for NAMR students introduced in the wake of the 2015 refugee situation formalized definitions and entitlements, and established clear guidelines for the assessment and reception of newly arrived pupils (Bunar, 2017; Mock-muñoz de Luna et al., 2019). Changes were based on evidence from a special commission entrusted with investigating the improvement of educational and integration outcomes for migrant pupils in Sweden (Bunar, 2017; Nilsson & Bunar, 2016). In terms of assessment, the new legislation introduced procedures and responsibilities for schools and education authorities receiving NAMR pupils, emphasizing ongoing assessment and mapping of previous skills and knowledge. In Malmö, this assessment and an introduction to the Swedish education system take place over a two-week period at the central reception centre in Malmö, a municipal agency. Recent evaluations indicate that while on paper these reforms represent an improvement, in practice their implementation is not yet systematic across school districts (Bunar, 2017). Schools in Sweden use introductory classes and direct integration, depending on a number of factors. Research suggests many introductory classes in Sweden continue to be located in physically separate facilities (Svensson, 2019).

#### Organizational aspect of education for newly arrived migrant students

Participating schools in Malmö had introductory classes, which interviewees described as being fully integrated within mainstream education. NAMR pupils in participating schools were expected to spend maximum 1 year in introductory classes, while simultaneously attending mainstream classes with their native-born peers. Mainstream classes consisted of a “home-room” type daily class, usually first thing in the morning, with a teacher acting as the main point of contact for all NAMR pupils in that class. Further mainstream subject classes could be attended according to NAMR pupils’ skills and competences. When deemed sufficiently proficient in Swedish, pupils transition fully to mainstream education, with continued language support.

One educator described the improved procedures for transitioning to mainstream education:

We also have a plan: one of the intro teachers who before did the testing will be a bridge between the introductory class and the ordinary class, so she can support the student when they start being part of the ordinary class. We also have a plan for the teacher with how they can adapt their education and their teaching so these children get the right materials and work in the ordinary class. (School teacher 2)

The teachers and headmasters from the four participating schools described access to resources for addressing health and wellbeing needs of their pupils, e.g., health personnel at the Student Health Team (EHT), and regular meetings health staff and teachers to discuss specific cases. Teachers knew how to reach them, and mentioned their competency in addressing the non-academic needs of newly arrived pupils, including health and wellbeing. The headmasters we interviewed said that collaboration among schools was encouraged by the national and local government. For example, the National School Agency provides materials and courses to support peer education.

All the teachers we interviewed described how the new guidelines/new policies for education of newly arrived students had improved the language and educational background assessment and ensured newly arrived students’ equal rights to accessing the national curriculum, after an initial focus on Swedish language in introductory classes, as illustrated by one teacher below:

[B]efore we didn’t know anything except their age and where they were from, at best. Now we know a little bit more, we know something about their school background and something about what they can do in reading, writing and numbers. (School teacher 2)

A number of teachers highlighted to shortage of mother-tongue tutors for NAMR pupils transitioning to mainstream school.

Some teachers considered that the new limits on students’ time in introductory classes results in insufficient preparation time for the transition to mainstream classes. In contrast, some interviewees thought this discourages students from getting “too comfortable” in introductory classes, and helps them integrate faster. In case of students with special learning needs, interviewees spoke of lacking resources, e.g., Swedish-as-a-second-language teachers. In one school, teachers explained that over 40 pupils transitioned to mainstream education per week, with only three staff available to accompany them. Another interviewee said that sometimes parents stepped in as student assistants when there was no time to arrange a professional assistant.

In conclusion, the interviewees agreed that the policy that was introduced in 2016 which regulates the specific rights of newly arrived students in compulsory school had indeed improved some structural aspects, but resources had not yet caught up. Teachers and headmasters thus felt that some organizational aspects of education for NAMR students prevented schools from fully meeting their needs. However, all the teachers seemed confident in their role teaching Swedish as a Second Language, and most expressed confidence in the support system at their schools, and when and how to access it. SLB framework describes how clear processes and accountability can reduce stress and increase confidence of front-line workers by reducing the “grey areas” and need for discretion in their daily work.

#### Problematization: teachers vs. headmasters’ views on the situation and needs of NAMR pupils

Educators interviewed in Malmö spoke of considering the “whole” individual in the school setting, i.e., pupils’ identity, skills, and wellbeing, as this interviewee confirmed:

… it’s not just an individual with a head, they have emotions and the whole lot, so that’s what we see, the whole individual in all aspects, both identity and health, wellbeing and skills, so we see that. (Municipal development secretary 1)

Two different perspectives emerged that seemed to contradict the “whole child” approach when considering the role of education and school in the health and wellbeing of NAMR children: Firstly, some teachers stressed the importance of pupils “feeling good” *in* school and leaving their private life at home, in what one interviewee referred to as a *salutogenic* approach. They feared knowledge about problems outside the classroom might interfere with their academic responsibilities and relationship with the pupil, evoking a don’t-ask-don’t-tell attitude among some of the interviewees:

[W]e don’t really feel that it would be a help for us to know their personal problems because probably it would affect me as well, as their teacher. (School teacher 1)

These educators seemed to limit “whole child” approach to a singular focus on the child as a learner, thus allowed them to maintain a limited definition of their role as teachers. This way of coping, i.e., narrowing their professional responsibilities and avoiding potential complexity and ambiguity, is described well in SLB research (Brodkin, 2011). It also highlights how teachers’ practices are shaped by the ways in which they problematize pupils and by the structural and the resource-related factors that impact their daily work. This approach is unfortunately not supported by the evidence presented above on how poor health and wellbeing may compromise children’s cognitive development (Konu & RimpelÄ, 2002; O’Connor et al., 2019). Furthermore, the approach reflects educators’ perceptions of being inadequately trained to deal with NAMR pupils’ often complex needs.

The second perspective reflects a more holistic view of NAMR pupils’ health and wellbeing, as exemplified by one school leader opinion that Swedish education- and child mental health services generally failed to address the needs of vulnerable children. She described her school lacked expertise on assisting children with debilitating conditions in the learning process. As she explained, by law children must attend school, but schools may not be able to address serious mental health needs:

… we have children who have a really, really big trauma and … who have a very, very bad psychic health. That’s also very difficult for us to work with because they have to go to school and there aren’t any other places to put them in that could work both on their mental health and their education at the same time … Those children, I feel that they are … we can’t meet them as we should. (School leader 1)

The same teacher envisioned a school system with integrated mental health provision to enable the inclusion of children in mainstream education, even if they have significant mental health issues:

[These children] are in the middle, between the health system and the school system, and I think there should be another system that [is] health system and school system together, to meet those children, to work with their mental health and their education side by side … (School leader 1)

#### Practices: educators’ strategies for addressing pupils’ health and wellbeing needs

Teachers and headmasters described discretionary and “coping” practices that were shaped in great part by the problematizations and structural features of their daily work.

In theory, all pupils, whether in introductory classes or not, can be referred to special needs support at any time during their education. However, different problematizations of learning difficulties and mental health for NAMR pupils, compared to their native peers, and the lack of valid assessment instruments resulted in psychologists’ reluctance to test the small percentage of pupils that present with potential learning difficulties. Educators explained that they continue to teach them to the best of their ability, even when they feel the pupil is not receiving adequate levels of support.

We have a bit of difficulty because the school psychologists don’t want to test newly arrived children because they think there are so many other things that can block the children and then the results are not confident, and then we cannot use them for getting some other special education. (School leader 2)

Creative approaches to learning, such as drama pedagogy, film and media, use of body language and images, were described as offering opportunities for different forms of self-expression and to narrate one’s story, and to express feelings. Furthermore, they were seen as ways of valuing pupils’ diversity and abilities, and thus promote their wellbeing through self-worth and validation.

So that you can show the baggage you carry in some way through multimedia, or different creative forms or- and that you sort of affirm; if the student has concentration problems; and vary accordingly. So what I do is more sensing the situation than actually talking about feelings, you try to vary and find different forms. (Municipal development secretary 1)

## Discussion

This study investigated the experiences and perceptions of teachers, headmasters and municipal workers regarding the health and wellbeing needs of NAMR children in Copenhagen, Denmark, and Malmö, Sweden, and the role of schools and educators in addressing these needs.

Most teachers and headmasters recognized that NAMR pupils faced unique health and wellbeing challenges related to their migration trajectory that may interfere with their learning abilities. However, while teachers in Copenhagen agreed that education and school settings played a role in addressing these needs, they expressed lacking resources and expertise to systematically do so, especially in the case of complex needs, e.g., trauma or migration-related issues. Several teachers and one school leader in Malmö echoed this opinion. Meanwhile, some Malmö educators viewed teaching as their sole responsibility and took a “don’t-ask-don’t-tell” stance regarding pupils’ family and personal background. However, they described relying to some extent on the school nurse services and other professionals within the school.

Educators in both settings mentioned a lack of validated screening tools blocked NAMR pupils’ access to mental health and special needs support. Furthermore, while recent policy changes to Swedish education clarified accountability and emphasized pupils strengths, recent policy changes in Denmark resulted in uncertainty and frustration among the educators we interviewed. In both settings, inflexible structural features of education for newly arrived students, limited resources and contradictory policy objectives, made some interviewees feel that they were committing “professional neglect” or “setting (their pupils) up to fail.” Interviewees in both settings highlighted the need for integrated, targeted mental health and psychosocial provision within schools.

Key obstacles to meeting the needs of NAMR pupils in Copenhagen were qualitative in nature, i.e., related to organizational structure, resources and educators’ training, while in Malmö obstacles included quantitative challenges related to the increase in NAMR pupils entering the education system. The Copenhagen education system received fewer NAMR children than anticipated.

The above findings should be viewed in light of a number of limitations. Firstly, a relatively small number of interviewees prevented us from comparing similarities or differences between the national contexts. The sample size reflected only the views of individual professionals in a small number of schools, in a highly decentralized national context. Secondly, focusing on two municipalities with high density of NAMR students and more experience in the field of education for newly-arrived children ignores the perspectives and experiences of educators in municipalities and schools with fewer migrant and refugee children. Thirdly, the application of the SLB theory does not necessarily account for the greater contextual factors, inside and outside the school setting, that influence how individual teachers and their headmasters perform their daily professional practices and develop their reflections on these practices. As a result, given the multiple and overlapping contexts, it is challenging to isolate the effect of certain policies on practices.

In terms of strengths, the comparative nature of the study allowed us to explore the perspectives and experiences of educators in two different Scandinavian settings with many shared characteristics, but divergent approaches to immigration and integration. The interviews provided a deeper insight into how national level education policies shaped the practices of teachers and schools at the local level, as well as the shared dimensions related to structural shortcomings in the provision for migrant and refugee pupils in both contexts.

In the wider context of national education policies, findings from both settings concurred to some extent with how each country approaches the integration of migrants in general, including the education of NAMR children. (Barcelona Centre for International Affairs [CIDOB], 2015; Mock-Muñoz de Luna, Hart, Krasnik et al., 2019). The vision of integration espoused by a given country is based on- and promotes specific perspectives of the nation, immigration and relationships between minority and majority populations (Rytter, 2018). The different approaches to migrant integration espoused in Denmark and Sweden to a certain extent shaped if and how the professionals in each setting were able to address the needs of NAMR pupils.

The impact of structural and organizational features of education on educators’ practices has been documented in SLB research. Those studies concur with our findings that structural features of education can reify existing educational and social inequities and create new ones (Brodkin, 2015; Musheno & Maynard-Moody, 2015; Svensson, 2019).

Teachers’ awareness and problematization of specific health and wellbeing issues faced by NAMR pupils in both settings concurred well with international findings (De Wal Pastoor, 2015; Fazel et al., 2012, 2005). Our findings also concurred with overwhelming evidence regarding teachers’ support for embedding mental health provision for migrant and refugee children within non-stigmatizing school environments, e.g., (Barghadouch et al., 2016; Ellis et al., 2011; Fazel et al., 2009; Pastoor, 2015). Our findings were in line with research highlighting the need for child-centred alternative ways of promoting health and wellbeing through meaningful activities, as expressed by education professionals, cautioning against the over-reliance on Western trauma-focused approaches (Block et al., 2014; Rutter, 2006).

SLB theory, as an analytical tool, sheds light on educators’ perspectives and experiences of working within the context of education for NAMR students in Copenhagen and Malmö—i.e., a highly politicized, and rapidly changing legislative and social landscape. Furthermore, SLB underscores the various paradoxes created by competing policy demands and dwindling resources. Education policies are important tools of welfare states and the precondition for their successful implementation is the reliance on “agents of the state” interpreting these policies, while socializing children into the welfare state principles and values (Tørslev & Børsch, 2017). Educators’ perspectives and experiences are therefore crucial to the refinement and effectiveness of these policies, and to achieving the general objectives of schools as welfare institutions in an equitable way.

Our study suggests that optimal, high-quality introduction education should be time-limited, and in units that are integrated in the school, thus allowing students to gradually increase their participation in regular classes with ongoing support. In the absence of such conditions, the transition from introductory classes to mainstream schooling can result in academic and social exclusion, as pupils feel out of their depth in the face of increasing academic demands with decreasing learning and social support (Nilsson & Axelsson, 2017).

Another paradox was that of the obstacles to the holistic child-centred approach. A number of educators in both settings spoke of embracing a “whole child” approach, which views children’s needs in a holistic way, yet expressed a) apprehension regarding discussing personal, not education-related topics with their NAMR pupils, or b) having no expertise in dealing with “war-trauma”. These contradictions reflected both the perceived lack of preparedness to deal with complex migration-related issues, as well as a fear of how it would affect the professionals on a personal level.

Equality in access to education for children residing in Scandinavian countries is a significant accomplishment towards fulfiling the human rights of children as enshrined in the United Nations Convention on the Rights of the Child (Assembly 1989), and according to Welfare State values (Telhaug et al., 2006). However, using the equity lens, the experiences and perspectives of a small sample of education professionals highlighted education system barriers to equity related to health and wellbeing needs of NAMR pupils in both settings. These findings demonstrated that education systems need to ensure that any specific needs impacting a child’s wellbeing and ability to learn, whether socio-economic or migration related, are recognized and targeted. Such a proposition would be in line with the Health in All Policies and intersectoral approaches which are gaining increasing traction in Scandinavia and elsewhere (Torgersen et al., 2007, Woodland et al., 2016).

Pulled in different directions, to the tune of diminishing resources, educators are a vital source of knowledge regarding how to address NAMR pupils’ needs, and the structural obstacles to equity in education. At a time when countries around the world are dealing with the challenges of integrating great numbers of newly arrived migrants, many of whom are refugees, national governments and societies would benefit from the inclusion of educators and migrant and refugee children and families in systemic deliberations on tackling inequity in educational and health outcomes between migrant (including refugee) and native born children.

## Supplementary Material

Supplemental Material
