# Supplementary material for: Towards more equitable education: meeting health and wellbeing needs of newly arrived migrant and refugee children—perspectives from educators in Denmark and Sweden
Source: Int J Qual Stud Health Well-being. 2020 Dec 9;15(Suppl 2):1773207. doi: 10.1080/17482631.2020.1773207 (PMC7733908; doi:10.1080/17482631.2020.1773207)
Supplement: Supplemental Material [file ZQHW_A_1773207_SM6780.docx]

**Supplementary materials**

***TABLE 1. Migrant education policies introduced in Copenhagen, Denmark since 2015***

| **COUNTRY** | **Type of policy** | **title** | **Last up-dated** | | **aim of policy** | **Population affected by policy** |
| --- | --- | --- | --- | --- | --- | --- |
| **denmark** | **LAWS** | 1. **Education Act** | | 2017 | To outline rights and access to primary and lower secondary education for all compulsory school-aged children legally residing in Denmark. | All children aged 6-16 who are legal residents of Denmark |
|  |  | 1. **Act on Special Municipal Programmes for Certain Immigrant Children and Youth** | | 2017 | In response to an increase in number of refugees arriving to Denmark, municipalities are allowed to set up and administer special education programmes as an alternative or supplement to existing reception classes.  Aim is for immigrant students to acquire the necessary personal, social and academic skills to participate in regular classes in compulsory or upper secondary education. For those above the age of compulsory education, it prepares them to find employment | Newly arrived immigrant and refugee children, aged 6-25. |
|  |  | 1. **Act on the Educational Environment for Students** | | 2016 | States the right of students to a healthy school environment, and obliges school leaders to compile an annual written evaluation of the school environment in terms of general safety and hygiene conditions as well as psychosocial and physical environment. | All children in compulsory education, aged 6-16 |
|  |  | 1. **Executive Order on Danish as a Second Language** | | 2016 | Expands the framework for reception of immigrant students in the education system: increase in maximum size of reception classes, more years/grade levels could be covered in reception classes. | Newly arrived immigrant students of compulsory school age. |
|  |  | 1. **Executive Order on the measurement of student well-being in primary and lower secondary schools** | | 2015 | Mandatory assessments of student wellbeing to be carried out in all Danish public schools, in order to inform the process of improving student wellbeing. The assessment aims to measure wellbeing along four dimensions: academic, social,  support and inspiration, peace and order, and general school wellbeing. | All students in Danish public schools in years 1-9.  Results are divided up according to immigrant background, i.e. immigrant, descendant and Danish origin. |

Danish migrant education policies introduced in the wake of the 2015 refugee situation further devolved migrant education to the municipal level, and expanded the framework for the reception of immigrant students in the education system in two ways. First by increasing the maximum number of students allowed in introductory classes from 12 to 15, and the number of grade levels covered from three to five, and second, by allowing municipalities to set up alternative or supplementary provision to the existing framework for reception classes, including special support for students with learning disabilities or mental health problems. Furthermore, these policy changes allowed provision that doesn’t comply with the Danish Education Act (which applies to all compulsory public education).

***TABLE 2. Migrant education policies introduced in Sweden since 2015***

| **COUNTRY** | **Type of policy** | **title** | **Last up-dated** | **aim of policy** | **Population affected by policy** |
| --- | --- | --- | --- | --- | --- |
| **Sweden** | **LAW** | **Education Act 2010, and subsequent amendments, 2016** | 2016 | Guarantees the right to primary and lower-secondary education to all children residing lawfully, as well as undocumented students. This law also provides the legislative framework for the education of newly arrived students, stating that they have the right to compulsory education, and assessment of their knowledge and skills as a basis for adapting education to their learning needs. Within the framework of the new legislation, municipalities are responsible for ensuring adequate provision, but schools are responsible for how they organize and structure introductory education and ongoing support for newly arrived students as long as it complies with the specifications laid out in the new legislation. | Compulsory for all children aged 7-16 living in Sweden, including asylum seekers and beneficiaries of international protection. Those with temporary residency are not subject to compulsory education but have a right to schooling. Asylum seekers and those with temporary residency are entitled to upper secondary education if enrolled before turning 18. |
|  | **GUIDELINE** | **Education for newly arrived students** | 2016 | Introduces more detailed guidelines, timeframes and accountability structures for the assessment of newly arrived students. Places great emphasis on the timely assessment of newly arrived students, to ensure they are placed in the appropriate provision as quickly as possible. Also requires ongoing assessment of students to ensure they can join mainstream education provision as soon as they are ready to do so. | Compulsory school aged children, 7-16. |

Legislative changes to migrant education introduced after 2015 provided a national legislative framework for the reception and education of newly arrived students into the education system, including clear definitions of who qualifies as a ‘newly arrived’ student, assessment procedures, and the type of support they are entitled to. In terms of newly arrived students’ assessment, the new legislation introduced procedures and responsibilities for schools and education authorities receiving newly arrived pupils, emphasizing thorough and timely ongoing assessment of newly arrived students and recognition of previous skills and knowledge to ensure their educational level and skills are adequately mapped.
